# Why do BCL-2 inhibitors work and where should we use them in the clinic?

**DOI:** 10.1038/cdd.2017.183

**Published:** 2017-10-27

**Authors:** Joan Montero, Antony Letai

**Affiliations:** 1 Department of Medical Oncology, Dana-Farber Cancer Institute, Harvard Medical School, Boston, MA 02215, USA

## Abstract

Intrinsic apoptosis is controlled by the BCL-2 family of proteins but the complexity of intra-family interactions makes it challenging to predict cell fate via standard molecular biology techniques. We discuss BCL-2 family regulation and how to determine cells’ readiness for apoptosis and anti-apoptotic dependence. Cancer cells often adopt anti-apoptotic defense mechanisms in response to oncogenic stress or anti-cancer therapy. However, by determining their anti-apoptotic addiction, we can use novel BH3 mimetics to overwhelm this apoptotic blockade. We outline the development and uses of these unique anti-apoptotic inhibitors and how to possibly combine them with other anti-cancer agents using dynamic BH3 profiling (DBP) to improve personalized cancer treatment.


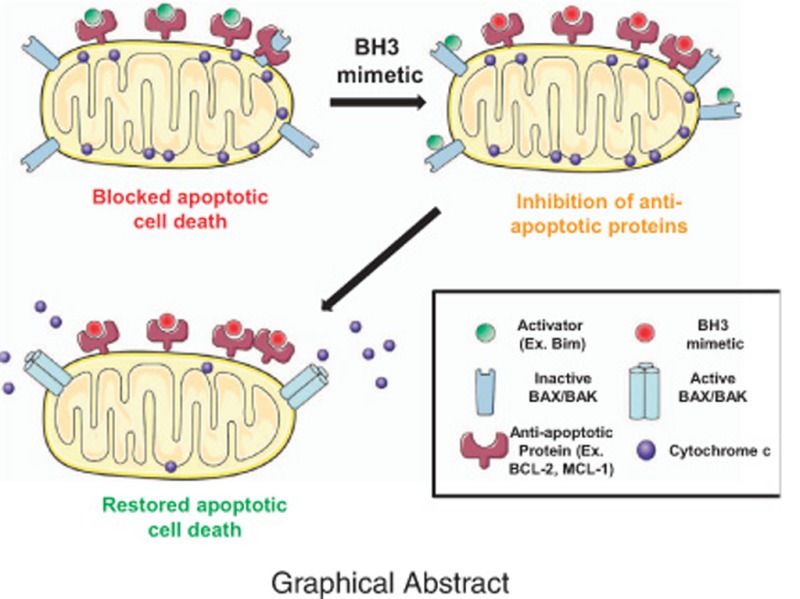


## Facts

- BCL-2 family proteins and their complex interactions regulate intrinsic apoptosis.
- BH3 profiling can determine cancer cells’ priming for cell death and anti-apoptotic dependencies.
- Cancer cells often acquire anti-apoptotic defense mechanisms against oncogenic stress and therapy.
- The DBP functional assay can be used to determine effective combinations of anti-cancer agents with BH3 mimetics to improve cancer treatment.

## Open Questions

- Is anti-apoptotic adaptation a common defense mechanism in most cancer types?
- Can BH3 mimetics alone or in combination be used to treat solid tumors?
- Will functional assays be able to guide BH3 mimetics’ use in the clinic?

Programmed cell death (PCD) has been observed in many forms of life from metazoans to mammals. Perhaps the best studied PCD pathway, and the first characterized, is apoptosis. Kerr and collaborators first described apoptosis in the early 70s in mammalian tissue sections where they observed that dying cells showed stereotypic nuclear condensation and cellular fragmentation. Moreover, they found that these fragments were phagocytosed by nearby cells.^1^ These fragments, also known as apoptotic bodies, are the remains of the plasma membrane, containing cell fragments and presenting phosphatidylserine in their surface as an 'eat me signal' that can be recognized by phagocytic white blood cells.^2^

Two distinct pathways of apoptosis have been previously characterized: extrinsic and intrinsic. The extrinsic pathway occurs when specific receptors on the cell surface called death receptors are activated, such as TNFR, FAS (CD95) and DR3/WSL. Ligand binding to the receptor induces a change in the intracellular region that promotes adapter proteins activation and death-inducing signaling complex formation. Thus, initiator caspases, such as caspase-8, get cleaved and activated, resulting in initiation of downstream executioner caspases that orchestrate apoptosis. Active caspase-8 also can cleave and activate the BH3-only protein BID that can promote the intrinsic pathway, connecting both apoptotic modes.^3^

Intrinsic apoptosis, also referred as the mitochondrial pathway, is executed in response to cellular damage and most anti-cancer agents, and the B-cell lymphoma 2 or BCL-2 family proteins regulate it. These proteins control mitochondrial outer membrane permeabilization (MOMP), which for most instances can be considered the point of no return for apoptosis. This permeabilization allows the release of soluble proteins such as cytochrome c and SMAC/DIABLO, from the mitochondrial intermembrane space into the cytosol. Once these proteins are released, cytochrome c binds to APAF-1 and caspase-9 in presence of dATP to form the apoptosome, which activates downstream effector caspases and triggers apoptosis.^4^

### BCL2: the family founder

The founding member of the family, *BCL-2* was identified as an oncogene resulting from a translocation between chromosomes 14 and 18 that promoted malignant lymphomagenesis.^5, 6^ In the early 90’s, several laboratories identified BCL-2 as a pro-survival protein that prevented apoptotic cell death and facilitated MYC-induced transformation.^7, 8^ The next obvious question at the time was: if BCL-2 protects from PCD, which proteins promote apoptosis? The answer came with the identification of a pro-death protein bound to BCL-2, named BCL-2-associated X or BAX, with similar structure and sequence homology to BCL-2.^9^ The other members of the so-called BCL-2 family were discovered in the following years based on protein interactions and their similar protein structure. They were classified as pro- or anti-apoptotic depending on their biological activity.^10, 11, 12, 13^ Following their identification, it became clear that MOMP and the release of cytochrome c was the triggering event of apoptosis and that BCL-2 prevented cell death by stopping this event.^14, 15, 16, 17^

### The BCL2 family of pro-apoptotic and anti-apoptotic proteins

The BCL-2 family proteins can be classified based on their structure and BCL-2 homology (BH) domains. The anti-apoptotic members BCL-2, BCL-XL, BCL-W, MCL-1 and A1/BFL-1 possess four BH domains, BH1-BH4, and present a hydrophobic groove in their structure that binds to the BH3 domain found in the pro-apoptotic. The pro-apoptotic effector proteins, BAX, BAK and BOK, possess three to four BH domains, and have the capacity to form pores in the mitochondrial outer membrane.^18, 19, 20^ These domains are composed of nine *α*-helices, including a transmembrane C-terminal *α*-helix that can anchor to the mitochondrial outer membrane. Their tridimensional structure are very similar to membrane pore-forming domains found in diphtheria toxin and colicins.^21^ There is a third group of proteins possessing only the BH3 domain, therefore named BH3-only proteins, with a pro-apoptotic function, including BIM, BID, PUMA, BAD, NOXA, HRK, BMF, BIK and others.^13, 22, 23, 24, 25, 26, 27^ Interaction between pro- and anti-apoptotic proteins takes place via the binding of the hydrophobic face of the amphipathic BH3 alpha-helix from the pro-apoptotic protein into a hydrophobic pocket in the anti-apoptotic protein formed by the BH1, BH2 and BH3 domains.

### Regulation of MOMP by the BCL-2 family of proteins

Once they are activated, effector proteins BAX and BAK oligomerize in the mitochondrial outer membrane to promote MOMP and cytochrome c release.^18, 19^ In fact, a double knockout of BAX and BAK prevents apoptosis in response to most stimuli.^28, 29^ The third effector protein BOK may be regulated by protein stability and can induce MOMP in response to endoplasmic reticulum stress insults, and its activation may not rely on other BCL-2 family proteins.^30^ However, regulation of BOK remains a matter of uncertainty.^31, 32^ BAX and BAK oligomerization can be directly engaged by activator BH3-only proteins such as BIM^13, 33^ and BID,^22^ although showing specificity differences.^34^ Other BH3-only proteins have recently emerged as direct activators as well, such as PUMA and NOXA, although with less potency compared to BIM or BID.^35, 36, 37, 38^ The BH3 domains found in these BH3-only proteins induce an allosteric change in BAX and BAK that promotes the formation of high-ordered oligomers that form pores in the mitochondrial outer membrane to cause MOMP.^39, 40, 41^

The anti-apoptotic proteins, including BCL-2, BCL-XL, MCL-1, BCL-W and BFL-1/A1, can prevent MOMP and apoptosis by two complementary mechanisms. They can directly bind the BH3 domain of activator BH3-only proteins, preventing BAX and BAK oligomerization.^42, 43, 44^ On the other hand, they can directly bind the activated monomeric forms of BAX and BAK, whose conformational change induces BH3 domain exposure.^45, 46, 47^ The most potent activators’ BH3 domains, like BIM, BID (and PUMA), interact promiscuously with all anti-apoptotic proteins and can directly activate BAX and BAK.

### The sensitizers within the family and their mechanism of action

**Figure 1:**
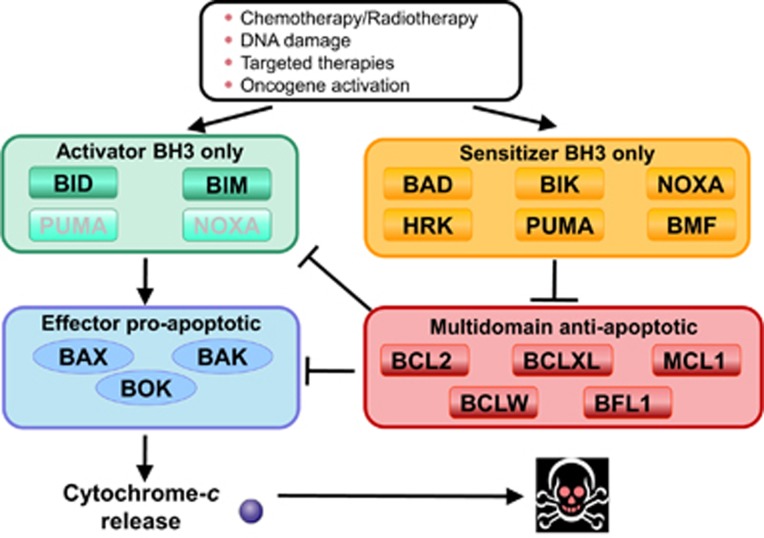
The BCL-2 family of proteins. BCL-2 family control over mitochondrial apoptosis representation. In response to therapy or oncogene activation, activators engage effectors, causing mitochondrial permeabilization and apoptotic cell death. PUMA and NOXA, are weaker activators compared with BIM or BID. Anti-apoptotic proteins sequester activators or effector proteins to prevent apoptosis. Sensitizers act as selective antagonists of anti-apoptotic proteins. Modified from Deng *et al.*, 2007

There is a fourth group of BCL-2 family proteins possessing only a BH3 domain, the so-called sensitizers. Sensitizers can be considered to include PUMA, BAD, HRK, NOXA, BMF and BIK. A pure sensitizer would not be able to directly activate BAX and BAK but rather exert pro-apoptotic effect by competing for binding in the hydrophobic pocket of the anti-apoptotic BCL-2 family members.^42, 44, 48^ It has become clear that there is something of a continuum between pure activator and pure sensitizer function, with BIM and BID being the most potent activators at one end, and others like PUMA possessing detectable but likely less-potent activity. In brief, the sensitizer BH3 domain binds to anti-apoptotic proteins, displacing activators that can activate the effectors and induce MOMP, exerting an indirect pro-apoptotic function.^42^ Alternatively, sensitizers can displace monomeric activated BAX or BAK that are sequestered by an anti-apoptotic protein. Sensitizers’ BH3 sequence governs their affinity for anti-apoptotic proteins. For example, BAD has high affinity for BCL-2, BCL-XL and BCL-W, but not MCL-1 or BFL-1. In contrast, HRK selectively binds to BCL-XL, and NOXA specifically binds to MCL-1 ^42^ (Figure 1). Therefore, the biological effect of sensitizers greatly depends upon the pre-existing balance of other BCL-2 family members at the mitochondria.^44, 49^

## Apoptosis, BH3 Mimetics and Cancer

### Are cancer cells more prone to apoptosis?

Why does chemotherapy work? Despite the broad use of anti-cancer agents in cancer treatment, and their ability to cure certain cancers, why chemotherapy selectively kills cancer cells and spares normal cells is poorly understood. Only recently Sarosiek and collaborators described how adult tissues are refractory for apoptosis, with low expression of apoptotic proteins in vital organs like brain, heart, kidney and liver, but not in hematopoietic tissues or intestine.^50, 51^ Chemo-sensitive cancer cells are more disposed for apoptosis than chemo-resistant cancer cells or normal cells, observations that likely elucidate why there is a therapeutic window to clinically treat tumors with chemotherapy.^50^

Cancer cells die by apoptosis when exposed to the right therapy. This apoptotic cell death can occur by inducing an up regulation of pro-apoptotic proteins or by directly decreasing the anti-apoptotic reservoir to allow the activators to initiate MOMP. Most anti-cancer agents like targeted therapies, DNA-damaging agents, microtubule inhibitors or other cytotoxic molecules, produce a cellular stress that leads to cell death.^52, 53^ This stress often primes by increasing expression of activators that can engage BAX/BAK to induce MOMP and apoptosis unless sequestered by anti-apoptotic proteins.^34, 52, 54, 55, 56, 57, 58^ Despite many successes in cancer therapy, many tumors are not efficiently killed by chemotherapy, leading some to suggest that cancer cells are generally blocked in apoptotic signaling.^51, 59^ It is important to ask, 'Compared to what?'. Many laboratories have observed that cells undergoing oncogenic transformation exhibit higher expression of pro-apoptotic proteins resulting from cell cycle checkpoint evasion, DNA replication stress, unfolded protein response or oxidative stress. However, cancer cells nonetheless survive by blocking these signals,^60, 61^ leaving a cell that is rather tenuously dependent for survival on continuation of that block. Rather than leaving a cell that is fundamentally prevented from undergoing apoptosis, very often the resulting cancer cell is quite ready to undergo apoptosis compared to normal cells. This distinction is, indeed, an important determinant of the therapeutic index for conventional chemotherapy that we have benefitted from for decades.^50, 62^

### Mechanisms of anti-apoptotic adaptation

One way that cancer cells can escape death from the pro-apoptotic signaling that emerges in oncogenesis or therapy is via selection for higher expression of anti-apoptotic proteins. Therefore, even if the cytotoxic insult is effective to induce a pro-death signaling by upregulating activator proteins like BIM or directly activating BAX or BAK, cancer cells can survive by sequestering them with anti-apoptotic proteins. This anti-apoptotic adaptation can vary from tumor to tumor, therefore it is necessary to evaluate it for every case.^49^ For instance, different cases of multiple myeloma (MM) can rely on BCL-2, BCL-XL, MCL-1 or combinations thereof for their survival, pointing to the need of case-specific treatments.^63^ In contrast, T-cell acute lymphoblastic leukemia anti-apoptotic dependency changes with the differentiation stage of the leukemic clone, relying largely on BCL-XL except for early T-cell progenitor cells that are BCL-2 dependent.^64^ Moreover, many laboratories have characterized tumor evolution and anti-apoptotic adaptation in response to therapy,^53, 65, 66, 67, 68, 69^ demanding rapid ways to measure it that will be discussed below. This state in which cancer cells constrain pro-apoptotic proteins via heterodimerization with anti-apoptotic proteins can be termed being ‘primed’ for apoptosis. In these cases, the anti-apoptotic defenses can be overwhelmed by blocking the anti-apoptotic proteins with specific inhibitors.^67, 70, 71, 72, 73, 74^

### Tackling anti-apoptotic adaptation

Consequently, great efforts have been directed towards finding small molecules to inhibit these anti-apoptotic BCL-2 family proteins and promote apoptosis in cancer with the so-called BH3 mimetics that mimic the action of certain BH3-only proteins. The first demonstration that abrogation of function of an anti-apoptotic protein by itself could lead to cancer regression was via the use of regulatable expression of BCL-2 in a murine model of leukemia.^75^ Later work found that deletion of MCL-1 caused regression of a similar MYC-driven lymphoma.^58^ The first attempt to inhibit the anti-apoptotic proteins was described in 2000 with the discovery of HA14-1, a small molecule targeting the BCL-2 surface pocket that showed activity *in vitro*.^76^ A subsequent effort by others produced obatoclax (GX-15-070), a drug that showed relatively low affinity,^77^ a BAX/BAK-independent induction of cell death ^78^ and modest clinical activity.^79^ Abbott Laboratories (Abbott Park, IL, USA) developed a small molecule matching the binding pattern of BAD, inhibiting BCL-2/BCL-XL/BCL-W, named ABT-737.^42, 80^ Preclinical studies with ABT-737 showed on-target activity and a requirement for BAX and BAK to induce apoptosis.^81^

Abbott Laboratories (now Abbvie) developed an orally bioavailable analog called ABT-263 (navitoclax) for clinical use. Preclinical studies with both ABT-737 and ABT-263 demonstrated their mechanism of action, displacing pro-apoptotic BH3-only proteins from BCL-2.^73^ When both agents were tested *in vivo*, a decrease in platelet counts owing to BCL-XL inhibition was observed, impairing ABT-263 use in the clinic.^70, 82, 83, 84^ However, ABT-263 related thrombocytopenia can be controlled using appropriate dosing^85, 86^ and several clinical trials evaluating its safety and efficacy are currently ongoing in CLL, small-cell lung cancer and other solid tumors^87, 88^ (see https://clinicaltrials.gov/ct2/results?term=abt-263). Encouraged by some striking responses in CLL patients to this BH3 mimetic, Abbvie developed an orally available inhibitor with 100-fold greater affinity for BCL-2 than BCL-XL, named ABT-199 (venetoclax).^89^ Owing to its high subnanomolar affinity for BCL-2 and low binding to BCL-XL, this novel agent does not cause thrombocytopenia. In fact, venetoclax was such a potent BCL-2 inhibitor that it caused tumor lysis syndrome in some CLL patients, imposing a dose-escalation strategy and the need for close surveillance in the clinic.^90^ The single-agent activity of venetoclax in relapsed CLL patients was outstanding with an 80% response rate and manageable secondary effects.^90^ Clinical trial results of venetoclax as a single agent led to its FDA approval in April 2016 for its use in CLL patients with 17p chromosomal deletion, a biomarker for TP53 loss and poor prognosis.^71, 90, 91^ Consequently, venetoclax became the first BH3 mimetic approved by the FDA for cancer treatment, encouraging many to follow.

Since then, a myriad of clinical trials started to evaluate this agent alone or in combination to treat multiple hematological malignancies (https://clinicaltrials.gov/ct2/results?term=abt-199). In contrast to relatively homogeneous results in CLL, other blood cancers like acute myeloid leukemia (AML), MM and non-Hodgkin’s lymphoma showed heterogeneous responses to single-agent venetoclax.^89, 92^ To improve response to this agent, many studies assessed its combination with other chemotherapeutic drugs. For instance, in AML the combination of venetoclax with hypomethylating agents results in an improvement of its clinical activity, achieving ~70% response rate (combined CR and CRi), in patients.^93^ In solid tumors, several reports showed *in vitro* response to venetoclax in breast^67^ and small-cell lung carcinoma lines,^80^ but only in certain cell lines, pointing to a need for stratification.

Following ABT-199 success, several pharmaceutical companies, including Servier, AstraZeneca and Amgen, are developing novel BCL-2, BCL-XL and MCL-1 inhibitors, and evaluating them in liquid and solid tumors,^94^ (https://clinicaltrials.gov/ct2/results?term=mcl-1+OR+bcl-2+OR+bcl-XL+inhibitor). Selective BCL-XL inhibition could be useful to treat certain types of tumors, as they upregulate it as a mechanism of protection against apoptosis.^95, 96^ However, BCL-XL dependence is found only in certain cases, pointing to an unmet need for predictive biomarkers for patient selection.^97^

There is evidence for the efficacy of MCL-1 inhibitors. However, although CLL is an example of a disease quite homogeneously dependent on BCL-2, a cancer homogeneously dependent on MCL-1 has yet to be identified. Heterogeneous MCL-1 dependence has been observed in non-small cell lung cancer lines, AML, chronic myelogenous leukemia, B-cell acute lymphoblastic leukemia (B-ALL) and MM.^66, 98, 99, 100, 101^ MCL-1 inhibition may have side effects like hematopoietic toxicity^102^ cardiotoxicity^103^ and perhaps affect mitochondrial respiration.^104^ However, it remains to be seen the extent to which titratable MCL-1 inhibition by drugs in humans phenocopies gene deletion in mice. As functions for MCL-1 not depending on BH3 domain binding have been identified, it is possible that some effects of MCL-1 deletion observed in mouse models may be separable from those caused by BH3 mimetic inhibition.^104^ Several MCL-1 inhibitors have been developed over the years using different strategies showing modest selectivity.^105, 106, 107^ However, the novel S63845 molecule developed by Servier exhibits exquisite selectivity for MCL-1 over BCL-2 and BCL-XL, and shows promising results.^72, 108^

Furthermore, based on the apoptotic resistance observed in vital organs in adults,^51^ there should theoretically be a therapeutic index to block all anti-apoptotic proteins by combining several BH3 mimetics. In fact, pre-clinical experiments showed that BCL-2/XL inhibition may lead to MCL-1 increased expression^65^ or BCL-XL^109, 110^ as a compensatory survival adaptation. Therefore, using a pan-BCL-2 inhibitor strategy to treat the most aggressive types of cancer may have clinical benefit. Like other chemotherapies used in the clinic, this therapeutic strategy would affect organs that are more ‘primed’ for apoptosis like the hematopoietic system, leading to well-characterized secondary effects. However, the clinical experience with myelosuppression lends optimism that hematologic toxicity can be managed via dosing, timing and combination of agents, and by closely monitoring patients. Moreover, should bone marrow toxicity be unavoidable, efficacious therapies can be made tolerable using autologous and allogeneic stem cell transplantation techniques. The clinical utility of these new inhibitors suggests that we will see an expansion of their use in the next years.

## Directing BH3 Mimetics in the Clinic

The dawn of BH3 mimetics brings an important question: how can we determine when to better use them to treat cancer patients? As discussed above, the BCL-2 family of proteins includes over a dozen members, each presenting different mode of regulation, post-translational modification, and protein–protein interactions, making difficult a correct prediction for BH3 mimetics’ response in cancer cells using standard molecular biology techniques. In other words, we have excellent agents to block anti-apoptotic defense in cancer cells but we lack efficient predictive biomarkers to guide their use. So far, most clinical successes with BH3 mimetics have arisen from exploiting lineage-based, rather than genome-based, vulnerabilities, so that genetic biomarkers have not been useful in predicting response. Therefore, there is a clear unmet need for novel ways to predict clinical efficacy of BH3 mimetics.

### Identifying cancer anti-apoptotic addiction through BH3 profiling

In this regard, the Letai laboratory developed a technique called BH3 profiling that can determine tumors’ dependencies on individual anti-apoptotic proteins.^42, 48, 111, 112, 113^ BH3 profiling expose cancer cells’ mitochondria to synthetic 20-mer BH3 peptides that mimic the pro-apoptotic function of BH3-only proteins to determine MOMP and apoptosis initiation.^44, 112, 113^ For instance, BIM and BID BH3 peptides interact with all endogenous anti-apoptotic proteins and can activate the effectors BAX and BAK, and are used as a measure of overall apoptotic priming.^34, 42, 51^ In contrast, the synthetic PUMA BH3 peptide binds with all the anti-apoptotic proteins but cannot activate BAX or BAK.^42^ The information obtained by BH3 profiling using these peptides is useful for identifying how close a cancer cell is to the apoptotic threshold, or how ’primed’ for apoptosis, and has proved to be a good predictor of response to conventional chemotherapy.^50, 51, 62^ Interestingly, certain BH3 peptides interact selectively with endogenous anti-apoptotic BCL-2 family proteins and can guide the therapeutic use of BH3 mimetics.^42, 48^ For example, MS1 and NOXA BH3 peptides interact selectively with MCL-1,^114^ the HRK BH3 peptide interacts selectively with BCL-XL, the BAD BH3 peptide interacts selectively with both BCL-2 and BCL-XL.^42^ By using these anti-apoptotic selective peptides, the Letai lab found BCL-2 dependence in CLL;^74^ heterogenous anti-apoptotic dependence in AML ^62, 115^ and MM;^63^ BCL-2 dependence in B-cell acute lymphoblastic leukemia and blastic plasmacytoid dendritic cell neoplasm;^73, 116, 117^ BCL-XL dependence in T-cell acute lymphoblastic leukemia, but BCL-2 dependence in early T-cell progenitor cell leukemia.^64, 118^

### Dynamic BH3 profiling (DBP)

**Figure 2:**
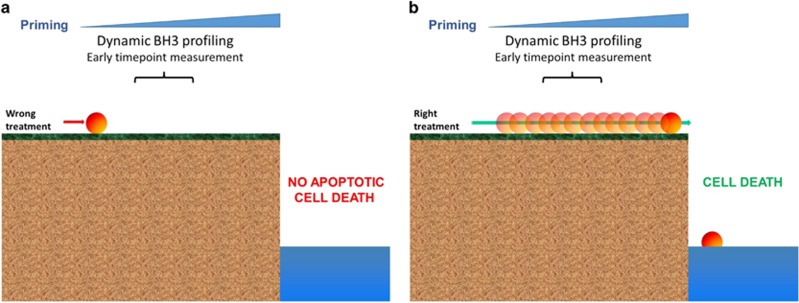
The threshold of apoptosis. We define priming as the proximity of a malignant cell to the threshold of apoptosis, and dynamic BH3 profiling (DBP) measures increases in priming upon treatment. (**a**) When a cancer cell is exposed to the wrong treatment there is no increase in priming, giving a negative result for DBP, and the cell will survive. (**b**) In contrast, when a cancer cell is exposed to the right treatment there is an increase in priming that will be detected by DBP in early timepoints preceding apoptotic cell death

Most anti-cancer agents, including kinase inhibitors, induce apoptosis in cells that are dependent on a pathway targeted by the inhibitor. When a cancer cell is treated with a targeted agent, early changes in the BCL-2 family of proteins can be rapidly detected preceding the activation of apoptosis. Taking advantage of this biological observation, the Letai laboratory developed a novel functional assay called DBP that measures net changes in mitochondrial apoptotic signaling in cancer cells following administration of targeted therapeutic agents using titrated doses of BIM BH3 peptide. DBP can reveal drug treatments that increase priming in cancer cells (termed Δ% priming), which is predictive of their later cell death *in vitro*, *in vivo* and directly in patient samples^119^ (Figure 2). This technology has been successfully applied to find new treatments for AML,^120^ B-ALL,^121, 122^ and to determine venetoclax response in blastic plasmacytoid dendritic cell neoplasm.^117^

## Combining BH3 Mimetics with Other Treatments

**Figure 3:**
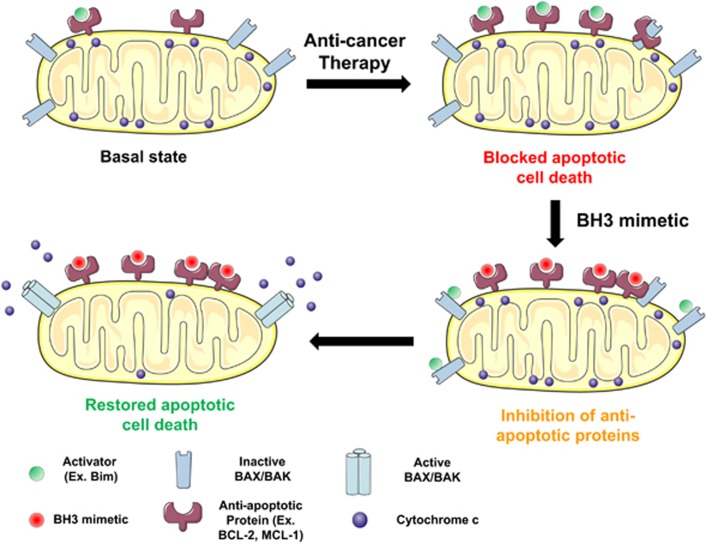
Use of BH3 mimetics to overcome tumors’ resistance to therapy. When cancer cells are exposed to therapy, they can adapt their anti-apoptotic strategy to block the pro-apoptotic proteins’ accumulation (activators and active effectors) induced by treatment. By blocking the anti-apoptotic defense using a specific BH3 mimetic, activators will be displaced from anti-apoptotic proteins and restore apoptotic cell death

Although we discussed clear examples in where BH3 mimetics can be useful as single agents to treat different types of cancer, many scientific reports suggest their therapeutic use in combination with other anti-cancer agents. For example, combining navitoclax with gemcitabine to treat solid tumors,^123^ navitoclax with brentuximab to treat Hodgkin’s lymphoma,^124^ JAK2 inhibitors with ABT-737^125^ or ABT-737 with cisplatin to treat non-small cell lung cancer.^126^ Particularly interesting are the possible combinations of targeted therapies with BH3 mimetics.^68, 126, 127, 128, 129^ In fact, dozens of clinical trials are currently evaluating the combined use of BH3 mimetics with other chemotherapeutic agents to treat multiple hematological malignancies (see clinicaltrials.gov). As discussed above, cancer cells’ anti-apoptotic dependence can alter when exposed to therapy by increasing the availability or upregulating anti-apoptotic proteins.^54, 56, 59^ By studying how this adaptation occurs, we can design novel therapeutic strategies combining anti-cancer agents and BH3 mimetics to restore cancer’s cell death (Figure 3).

**Figure 4:**
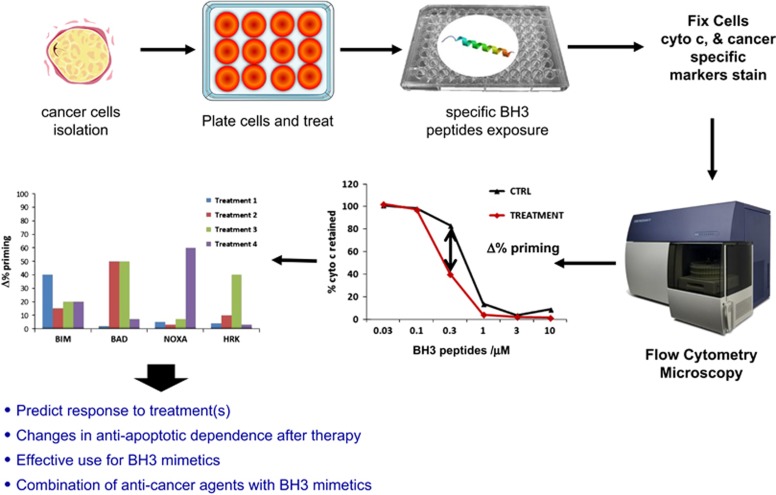
Using dynamic BH3 profiling to identify BH3 mimetics’ use. To perform dynamic BH3 profiling, we obtain a single cell suspension from a cell line or a primary sample, and we expose the cells for a short incubation with the different treatments of interest. After this incubation, we permeabilize cells with digitonin, and expose the cells to selective BH3 peptides. We then analyze cytochrome c retention in the cells by flow cytometry or microscopy. By comparing non-treated with treated cells, we will determine the Δ%priming for each agent and identify which are most effective to induce apoptosis or detect anti-apoptotic dependency changes. BIM BH3 peptide will indicate overall response to treatment (treatment 1). A BAD BH3-positive signal and negative HRK signal will indicate BCL-2 dependence and a possible use for agents like ABT-199/venetoclax (treatment 2). HRK-positive signal will indicate BCL-XL dependence and possible use for inhibitors against this antiapoptotic protein (treatment 3). NOXA-positive signal will indicate the use of specific MCL-1 inhibitors (treatment 4). Adapted from Montero *et al.*, 2015 and Ryan *et al.*, 2016

This brings us to our initial question: how can we determine when to use BH3 mimetics, and in what combinations, to treat cancer patients? In this regard, we can perform DBP, challenging cancer cells with active agents of interest, and then use specific peptides like BAD, HRK or NOXA to determine increased dependence on BCL-2, BCL-XL or MCL-1. We can analyze this information to design rational therapeutic strategies exploiting cancer’s anti-apoptotic vulnerabilities. For example, we can examine combinations of agents like kinase inhibitors or conventional chemotherapy with BH3 mimetics to treat cancer patients. These drugs can be used together, or be given sequentially in a metronomic regime to increase potency and minimize secondary effects, and DBP can evaluate many possibilities to guide precision medicine (Figure 4). In fact, this strategy has already been successfully applied in chronic lymphocytic leukemia patient samples by combining the BTK inhibitor ibrutinib, which led to increased BCL-2 dependence, with venetoclax to enhance cancer cells’ killing.^127^ We and others believe that the next years will witness an expansion of similar strategies to treat different types of cancer, both liquid and solid.

## Future Perspectives

As discussed above, many reports showed that cancer cells often present anti-apoptotic adaptation to ensure survival against oncogenic stress or anti-cancer therapy. Even if this adaptation has been observed in many malignancies, it is still unknown if this is a common feature for all human cancers or if on the contrary it is observed more heterogeneously. With FDA-approval of venetoclax in CLL, many oncologists and cancer researchers are exploring the uses of BH3 mimetics in other contexts in the clinic. Particularly, in hematological malignancies a myriad of clinical trials are assessing the possible use of venetoclax alone or in combination with other chemotherapies. Furthermore, several pharmaceutical companies are developing other BH3 mimetics targeting not only BCL-2, but also BCL-XL and MCL-1. It remains to be seen whether the excellent results observed in diseases like CLL using anti-apoptotic inhibition will be mirrored in solid tumors that are commonly more difficult to treat in the clinic. The authors expect that a diversity of BH3 mimetics will need to be employed, with likely a greater importance for BCL-XL inhibition in solid tumors than has so far been observed in hematologic tumors. One of the biggest problems for the use of BH3 mimetics is the lack of specific biomarkers to guide their use. We believe that functional assays like BH3 profiling or DBP may be useful to determine their best use. Undoubtedly, the use of BH3 mimetics in the clinic represents the dawn of a new era for personalized cancer treatment with great promise to improve patients’ clinical outcome.
